# Pancreatic Adenocarcinoma Complicated by Sinistral Portal Hypertension

**DOI:** 10.7759/cureus.689

**Published:** 2016-07-14

**Authors:** Muhammad W Saif, Kristin Kaley, Lynne Lamb

**Affiliations:** 1 Hematology/Oncology, Tufts Medical Center; 2 Yale School of Medicine, Yale University

**Keywords:** deep venous thrombosis, splenic hypertension, portal hypertension, non-cirrhotic portal hypertension, variceal bleed

## Abstract

Pancreatic cancer is known for vague symptoms that lead to a delay in diagnosis, and hence most cases are found at an advanced stage. Many complications can happen secondary to pancreatic cancer including diabetes, malabsorption, and deep venous thrombosis. Sinistral (segmental or left-sided) portal hypertension (SPH) refers to portal hypertension confined to the left-sided segment of the portal venous system namely the splenic side, and the most common etiology is splenic vein thrombosis (SVT). We present here a case of a 66-year-old male with advanced pancreatic cancer who died due to bleeding secondary to SVT. We advise physicians caring for these patients to be aware of this complication, which may also be the manifestation of an undiagnosed pancreatic cancer.

## Introduction

Pancreatic cancer is known for presenting with nonspecific symptoms, leading to delay in earlier diagnosis. The most common symptoms include weight loss, abdominal pain, jaundice, and anorexia. Gastrointestinal bleeding (GIB) is a known complication seen in patients with pancreatic cancer, especially if the tumor of the head of the pancreas perforates into the duodenum. The less frequent cause of GIB in these patients includes gastrointestinal hemorrhage through inflammation, postoperative complications, radiation complication, radiation recall phenomenon associated with certain chemotherapy agents, and congenital abnormality [1-2]. SVT is a known complication of pancreatic cancer that may lead to GIB [3]. We present here a case of GIB secondary to SPH resulting from SVT as a complication of pancreatic cancer.

Informed consent was obtained from the patient for this study.

## Case presentation

A 66-year-old male with a past medical history of stage IV pancreatic cancer with metastasis to the liver, who was on chemotherapy treatment consisting of gemcitabine and an oral experimental agent, inosine monophosphate dehydrogenase (IMPDH), presented with increasing abdominal pain and nausea to the emergency department. The patient reported that abdominal pain increased after biliary stent replacement four days ago. Though the patient used oxycodone with dilaudid at home for pain control, he reported that the pain was increasing. On admission, he graded the pain as 8/10. The patient also complained of constipation. His last bowel movement was several days prior to his admission. The patient denied any fevers, chills, or any kind of urinary symptoms.

His medications included pancreatic enzyme replacement, omeprazole, levothyroxine, sodium docusate, and pain medications as mentioned above. He admitted to drinking alcohol socially and denied smoking history. The patient was started on intravenous dilaudid and the pain improved. During his hospital stay, the belly remained distended and tympanitic, which was attributed to constipation due to the initial high doses of narcotics. The patient's abdominal x-ray subsequently showed an ileus with improvement during his hospitalization. His laboratory results showed that he was clostridium difficile antigen positive with clostridium difficile toxin negative and he was placed on intravenous (IV) metronidazole. However, on day 10 of hospitalization, the patient had an episode of nausea followed by emesis of 100-200 cc of bright red blood. He had passed brown stool and also complained of mid epigastric abdominal pain. On further evaluation, he was found to be orthostatic.

Gastroenterology service was consulted and the patient was transferred to the intensive care unit for further management. An upper endoscopy revealed large varices with nipple and red wale signs at the gastroesophageal (GE) junction. Though the varices were banded, a large amount of blood was seen in the stomach. An axial IV enhanced computerized tomography (CT) scan of the abdomen and pelvis was repeated and it showed a pancreatic mass extending into the retroperitoneum and surrounding the hepatic artery, splenic artery, and celiac axis measuring 3.1 cm, which was unchanged. Multiple liver metastases showed an overall improvement. A CT scan also showed an insertion of the internal biliary stent with a resolution of intra and extrahepatic biliary duct dilatation, development of pneumobilia in the gallbladder, pancreatic and peripancreatic edema, and swelling in the background of the infiltrative pancreatic tumor with the suggestion of pancreatitis and minimal ascites. Adrenal glands and kidneys are unremarkable with the exception of renal cysts. Incidentally, a new splenic vein thrombosis and mild splenomegaly were found. Amylase and lipase levels were within normal range. Liver function tests remained stable (total bilirubin 1.2 mg/dL; AST 66 IU/L; ALT 178 IU/L and alkaline phosphatase 347 IU/L). An abdominal ultrasound was performed and it confirmed the findings of the CT scan, specifically the SVT.

The patient continued to bleed, requiring multiple units of red blood cells. Interventional radiology was consulted for the possibility of an angiogram and embolization. The decision to give the patient comfort care was made at a family meeting. The patient was then transferred out of the intensive care unit to the oncology service with the goal of care for comfort. The patient was being treated with dilaudid and lorazepam under the supervision of palliative care team. Sadly, six days later the patient passed away, but he remained comfortable throughout the course of his hospitalization. An autopsy was offered but was denied by the family.

## Discussion

SPH resulting from SVT secondary to pancreatic adenocarcinoma is presented here as a cause of GIB. SPH is an extremely rare syndrome with an incidence of less than one percent, however, under recognition, may be a factor in the available reported low incidence [4]. SPH is defined as a clinical syndrome in which the thrombosis of the splenic vein manifests itself with bleeding gastric varices in a patient with a patent portal vein and normal hepatic functions evidenced by the liver function tests (LFTs) [4-5]. The term "sinistral" portal hypertension is employed to indicate the segmental or only left-sided involvement of the portal venous systems (mainly splenic vein), causing portal hypertension.

The most common etiologies associated with SVT include pancreatic cancer, chronic pancreatitis, pancreatic pseudocyst, trauma, pancreatic tuberculosis, retroperitoneal fibrosis, peptic ulcer disease, or a hypercoagulable state [6-8].

SVT by itself manifests with the triad of isolated gastric varices, splenomegaly, and normal hepatic function. In the majority of the patients, bleeding is the most common presentation associated with this syndrome, however, SPH can occur without bleeding. In addition, SVT can also occur without evidence of SPH. SPH resulting from splenic vein thrombosis is a recognized complication of pancreatic cancer. A review of the literature suggests that most cases present with a primary location in the tail of the pancreas, while our patient had it primarily in the body of the pancreas.

The anatomy of the pancreas and its surrounding structures and vessels can explain the underlying pathogenesis. The splenic vein runs in close proximity to the entire length of the pancreas, and any process occurring in the pancreas, be it cancer or inflammation, may result in spasm or compression of the splenic vein, thereby causing stasis, leading to intimal damage and development of thrombosis [10]. Hence, any blockage of the splenic vein could cause pooling of blood and subsequently produce high pressure upstream, particularly on the left side of the portal venous circulation and then result in SPH [9].

The diagnosis of SVT is usually confirmed with a splenic angiogram, however, other imaging modalities such as CT scan as well as ultrasound may also demonstrate a splenic vein thrombus. Splenectomy is considered to be the best treatment in ameliorating bleeding gastric varices in these patients. However, the literature also suggests that splenectomy may not be the ideal option in certain patients as this procedure may exacerbate gastric variceal bleeding. In such cases, supportive measures may be indicated.

A review of the literature also showed one case where SPH and SVT were the first sign of pancreatic cancer, alerting us to the fact that we need to be aware of this entity associated with pancreatic cancer. The authors suggested that pancreatic cancer should be suspected in patients with SPH [10].

## Conclusions

Physicians caring for SPH patients with SVT need to be aware that this is a known complication of pancreatic cancer. In addition, an underlying pancreatic cancer should be suspected in a patient who has SPH. Selected cases can benefit from splenectomy and distal pancreatectomy, however, others may only receive supportive measures including management of gastric bleeding and blood transfusions.
